# Paclitaxel-Coated Balloon Angioplasty for the Treatment of Infrainguinal Arteries: 24-Month Outcomes in the Full Cohort of BIOLUX P-III Global Registry

**DOI:** 10.1007/s00270-020-02663-7

**Published:** 2020-10-20

**Authors:** Gunnar Tepe, Thomas Zeller, Matej Moscovic, Jean-Marc Corpataux, Johnny Kent Christensen, Koen Keirse, Giovanni Nano, Henrik Schroeder, Christoph A. Binkert, Marianne Brodmann

**Affiliations:** 1 Department of Radiology, Klinikum Rosenheim, Rosenheim, Germany; 2 Clinic Cardiology and Angiology II, Universitäts-Herzzentrum Freiburg – Bad Krozingen, Bad Krozingen, Germany; 3 Angiology Clinic, Institute of Cardiovascular Diseases, Kosice, Slovakia; 4 Department of Vascular Surgery, Lausanne University Hospital, Lausanne, Switzerland; 5 Department of Radiology, Kolding Hospital, Kolding, Denmark; 6 Department of Vascular Surgery, Regional Hospital Heilig Hart, Tienen, Belgium; 7 1st Vascular Surgery Department, IRCCS Policlinico San Donato, San Donato Milanese, Italy; 8 Center for Diagnostic Radiology and Minimally Invasive Therapy, Jewish Hospital, Berlin, Germany; 9 Radiology Institute, Kantonsspital Winterthur, Winterthur, Switzerland; 10 Division of Angiology, Department of Internal Medicine, Medical University Graz, Graz, Austria; 11 Institut für Diagnostische Und Interventionelle Radiologie, RoMed Klinikum Rosenheim, Pettenkoferstr. 10, 83022 Rosenheim, Germany

**Keywords:** Drug-coated balloon, Peripheral artery disease, Paclitaxel, Femoral artery, Critical limb ischemia

## Abstract

**Purpose:**

After promising small randomized trials, the aim of BIOLUX P-III was to further investigate the safety and performance of the Passeo-18 lx drug-coated balloon in infrainguinal arteries under real-world conditions.

**Methods:**

BIOLUX P-III is a global prospective single-arm study with follow-up at 6, 12 and 24 months. The primary safety endpoint was freedom from major adverse events (MAE) within 6 months. The primary performance endpoint was freedom from clinically driven target lesion revascularization (TLR) within 12 months.

**Results:**

877 patients/1084 lesions were enrolled. Diabetes mellitus was present in 47.7%, and 42.1% had critical limb ischemia (CLI). The mean lesion length was 89.0 mm with 76.1% of calcified lesions, and 24.9% occluded. At 24 months, freedom from MAE was 83.1% in the full cohort; 84.9% in the femoropopliteal population (592 patients, 691 lesions); 77.7% for long lesions (187 subjects/192 lesions); and 72.5% in the in-stent restenosis (ISR) subgroup (103 subjects/116 lesions). Twenty-four-month freedom from clinically driven TLR was 88.1% in the full cohort; 88.9% in the femoropopliteal population; 80.3% for the long lesions; and 78.4% for ISR. Twenty-four-month all-cause mortality was 12.0% in the full cohort, 10.2% in the femoropopliteal population, 14.8% for the long lesions and 12.0% for ISR. There was no device- or procedure-related death up to 24-month follow-up.

**Conclusion:**

The BIOLUX P-III 24-month outcomes confirm the safety and performance of Passeo-18 lx in infrainguinal arteries in a large population treated under real-world conditions with low complication rates and good clinical outcomes (NCT02276313).

**Electronic supplementary material:**

The online version of this article (10.1007/s00270-020-02663-7) contains supplementary material, which is available to authorized users.

## Introduction

Lower extremity artery disease is present in approximately 202 million people worldwide (approximately 40 million in Europe) [1]. Previously treated by surgery, endovascular therapy is meanwhile used in the majority of lesions. While standard balloon angioplasty alone is limited by low long-term patency, stents can improve patency. However, the long-term durability of stent is also challenged, in particular, in very mobile arterial segment such as in the femoropopliteal artery. Moreover, treatment of in-stent restenosis (ISR) is more challenging than revascularization after balloon angioplasty [1]. But none of these techniques is sufficient enough from a biological point of view to prevent recurrent stenosis due to smooth muscle cells proliferation and migration, extracellular matrix production and finally neointimal hyperplasia.

Following numerous randomized controlled trials (RCT) showing superior outcomes for drug-coated balloon (DCB) over uncoated balloons [2–7], DCB is broadly used as a first-line therapy in femoropopliteal lesions [8]. For the treatment of below-the-knee artery lesions, the outcomes have been controversial so far [9, 10].

The aim of the BIOLUX P-III all-comers single-arm study was to strengthen Passeo-18 lx promising efficacy and safety outcomes shown in BIOLUX P-I and BIOLUX P-II first-in-human RCTs [2, 9] in a large real-world patient population with long-term safety data. Twelve-month results of the first 700 patients, namely the all-comers cohort, have been published [11]. We now report the 12- and 24-month outcomes of the full cohort and 3 selected predefined subgroups: femoropopliteal lesions, long lesions, ISR.

## Methods

### Study Design and Patient Population

The study has been previously described [11, 12]. The aim of this prospective, non-randomized, multicenter, international, observational single-arm study was to evaluate the safety and clinical performance of the Passeo-18 lx DCB in a large unselected patient population, including below-the-knee lesions and Rutherford 5 or 6, under real-world conditions. Seven hundred subjects were scheduled to be enrolled in this study, allowing for an increase in sample size to reach pre-specified subgroup sizes. Enrolment of the first 700 patients, namely the all-comers cohort, occurred from October 2014 to February 2016. The all-comers cohort which represents a sample of consecutive patients treated with the Passeo-18 lx DCB was expanded to 877 patients (full cohort) treated until January 2017 to reach the minimum number of subjects in pre-defined subgroups. This report includes the 24-month results for the full cohort, with a focus on femoropopliteal lesions, long lesions (15 cm or greater) and ISR.

Patients were eligible if they had lesions in the infrainguinal arteries suitable for endovascular treatment with the Passeo-18 lx DCB. Excluded were patients with a life expectancy of less than one year, or failure to successfully cross the target lesion with a guide wire.

The procedure, follow-up assessments and antiplatelet therapy were at the investigators’ discretion and according to standard of care at the study centers. Data were collected at baseline/intervention, at discharge, and at 6-, 12- and 24-month follow-up visits. The lesion characteristics were estimated visually by the investigator. The degree of calcification was assessed according to semi-quantitative categories, using the approach suggested by Diehm et al. [13]. No core laboratory assessment was performed. The visit windows were ± 30 days for the 6-month and ± 60 days for the 12- and 24-month follow-up visits.

The study was conducted according to the Declaration of Helsinki and ISO14155:2011 as applicable and approved by the relevant ethical review board affiliated with each participating center. All patients provided written informed consent. To ensure data quality, a risk-based monitoring approach was applied. At least 25% of the subjects enrolled at each site were randomly chosen and fully monitored (100% source data verification) on site. When less than 4 patients were enrolled by a site, at least one patient was fully monitored. Besides, all target lesion revascularizations (TLR), major adverse events (MAE) and deaths were adjudicated by an independent clinical events committee (CEC). The study is registered at clinicaltrials.gov (NCT02276313).

### Study Device

The Passeo-18 lx DCB (BIOTRONIK AG, Switzerland), CE marked since 2014, has been previously described [2, 9]. In brief, it is a balloon that is coated with 3 µg paclitaxel per mm^2^ incorporated in the excipient Butyryl-tri-hexyl-citrate (BTHC). Sizes available were 2.0–7.0 mm diameter and lengths of 40, 80 and 120 mm.

### Study Endpoints

The primary safety endpoint is freedom from MAE, a composite of freedom from device- and procedure-related mortality through 30 days, freedom from major target limb amputation and clinically driven TLR within 6 months post-index procedure. The primary performance endpoint is freedom from clinically driven TLR within 12 months post-index procedure. Thereby, clinically driven TLR is defined as any re-intervention performed for ≥ 50% diameter stenosis (visual estimate) at the target lesion after documentation of recurrent clinical symptoms of the patient.

Secondary endpoints are freedom from clinically driven TLR at 6 and 24 months post-index procedure, freedom from clinically driven target vessel revascularization (TVR) and amputation free survival at 6, 12 and 24 months (a composite of target limb major amputation and death), primary patency (defined as freedom from > 50% restenosis in the target lesion as indicated by a duplex ultrasound peak systolic velocity ratio (PSVR) > 2.5 or by visual assessment of an angiogram with no clinically driven re-intervention), and freedom from MAE at 12 and 24 months. Primary patency and clinically driven TLR were evaluated on a per lesion basis. Additionally, the mean Ankle Brachial Index (ABI) and patient-reported outcomes such as pain scale and walking impairment questionnaires had to be documented at each follow-up visit.

### Statistical Analysis

Considering the observational study design, BIOLUX P-III does not involve a hypothesis-driven sample size estimation. The overall sample size for this observational study was determined to ensure a sufficient number of patients in the predefined subgroup. Numerical variables are presented as mean ± standard deviation. Categorical variables are presented as frequencies and percentages of the total. Clinical outcomes were calculated using the Kaplan–Meier methods and standard error for the survival estimators was calculated using the Greenwood's formula. Confidence intervals were calculated as appropriate. Comparisons to baseline variables were conducted using the t test, Wilcoxon signed-rank test or exact sign test. All tests were performed at 95% confidence level.

A multivariate Cox regression analysis was performed to identify whether a relationship between mortality and paclitaxel exposure during the index procedure could be identified, taking potential confounders into account. The drug load per balloon size is described in Passeo-18 lx instruction for use. The paclitaxel exposure per patient was defined by adding the drug load on each balloon used during the index procedure.

All statistical analyses were carried out using SAS 9.4 (SAS Institute Inc. Cary, NC, USA).

## Results

The study was conducted at 44 centers in 16 countries in Europe, Asia and Australia (Supplemental online Table 3). The full cohort comprises 877 patients enrolled between October 2014 and January 2017. Mean age was 70.1 ± 10.2 years, and 64.0% of patients were male. 57.5% of the full cohort had a history of peripheral artery disease (PAD) with previous peripheral procedure or surgery performed in 49.9%. Approximately half of the patients had diabetes, 42.1% had CLI (critical limb ischemia) and about one-third of patients had renal disease (Table 1). A total of 1084 lesions were treated, thereof 75.4% affected femoropopliteal arteries and 17.0% infrapopliteal arteries. More than half of the lesions were de novo lesions (54.1%), 24.9% were occluded and 10.7% were in-stent restenosis. Three quarters of the lesions were calcified, 44.6% moderately or heavily calcified, and 32.6% of lesions were TASC C/D lesions. The mean target lesion length was 89.0 ± 77.0 mm and mean reference vessel diameter 4.7 ± 1.1 mm (Table 2).

**Table 1:** Baseline demographic characteristics

	Full cohort *N* = 877 patients	Femoropopliteal^a^ *N* = 592 patients	Long lesion (≥ 15 cm) *N* = 187	In-stent restenosis *N* = 103
Male	64.0% (561/877)	62.7% (371/592)	58.8% (110/187)	64.1% (66/103)
Age (years)	70.1 ± 10.2 [69.5, 70.8]	69.5 ± 10.2 [68.7, 70.3]	69.8 ± 10.1 [68.4, 71.3]	70.4 ± 9.8 [68.5, 72.3]
Body mass index (kg/m^2^)	26.9 ± 4.3 [26.6, 27.2]	26.9 ± 4.4 [26.5, 27.2]	26.4 ± 3.8 [25.8, 27.0]	27.2 ± 3.9 [26.3; 28.0]
Hypertension	84.8% (744/877)	84.3% (499/592)	87.2% (163/187)	89.3% (92/103)
Hyperlipidemia	67.1% (588/876)	68.2% (403/591)	68.4% (128/187)	82.5% (85/103)
Diabetes	47.7% (418/877)	42.9% (254/592)	45.5% (85/187)	42.7% (44/103)
History of peripheral arterial occlusive disease	57.5% (504/877)	58.6% (347/592)	65.2% (122/187)	91.3% (94/103)
Smoking habits				
Never smoked	32.1% (280/872)	25.7% (152/592)	28.9% (54/187)	18.6% (19/102)
Smoker	67.9% (592/872)	74.3% (440/592)	71.1% (133/187)	81.4% (83/102)
Ex-smoker	61.5% (364/592)	59.5% (262/440)	60.2% (80/133)	68.7% (57/83)
Current-smoker	38.5% (228/592)	40.5% (178/440)	39.8% (53/133)	31.3% (26/83)
Renal disease (insufficiency)	35.8% (314/877)	32.8% (194/592)	33.7% (63/187)	40.8% (42/103)
Dialysis	14.0% (44/314)	10.3% (20/194)	15.9% (10/63)	7.1% (3/42)
Coronary artery disease	42.0% (368/877)	41.9% (248/592)	39.0% (73/187)	42.7% (44/103)
Cerebrovascular disease	19.1% (167/876)	19.1% (113/591)	21.4% (40/187)	16.7% (17/102)
Subjects with previous peripheral interventions/surgeries	49.9% (438/877)	53.7% (318/592)	52.9% (99/187)	98.1% (101/103)
Cancer	11.4% (100/877)	14.0% (75/536)	9.1% (17/187)	15.5% (16/103)
Rutherford classification	*N* = 779	*N* = 538	*N* = 179	*N* = 85
Category 0	0.1% (1/779)*	0% (0/538)	0% (0/179)	0% (0/85)
Category 1	1.5% (12/779)	1.5% (8/538)	1.1% (2/179)	4.7% (4/85)
Category 2	14.9% (116/779)	17.3% (93/538)	14.0% (25/179)	20.0% (17/85)
Category 3	41.3% (322/779)	46.3% (249/538)	53.1% (95/179)	36.5% (31/85)
Category 4	13.2% (103/779)	13.0% (70/538)	5.6% (10/179)	17.6% (15/85)
Category 5	21.6% (168/779)	17.5% (94/538)	20.1% (36/179)	16.5% (14/85)
Category 6	7.3% (57/779)	4.5% (24/538)	6.1% (11/179)	4.7% (4/85)
Limited by contralateral limb	19.9% (155/779)	21.0% (113/538)	17.9% (32/179)	24.7% (21/85)

Continuous data are presented as the means ± standard deviation and [95%CI]; categorical data are given as the *n*/*N* (percentage)

^a^Superficial femoral artery and proximal popliteal artery. (*) Preventive treatment of in-stent restenosis to prevent stent closure

**Table 2:** Baseline lesion characteristics

Lesions	Ful cohort *N* = 1084	Femoropopliteal lesions^a^ *N* = 691	Long lesions (≥ 15 cm) *N* = 192	In-stent restenosis *N* = 116
Indication				
De-novo lesion	54.1% (586/1084)	51.5% (356/601)	35.9% (69/192)	0% (0/116)
Re-stenosis	10.3% (112/1084)	9.8% (68/691)	6.3% (12/192)	0% (0/116)
In-stent re-stenosis	10.7% (116/1084)	13.5% (93/691)	11.5% (22/192)	100% (116/116)
Occlusion	24.9% (270/1084)	25.2% (174/691)	46.4% (89/192)	0% (0/116)
Mean target lesion length (visual estimate) (mm)	89.0 ± 77.0 [84.4, 93.6]	96.2 ± 78.2 [90.3, 102.0]	227.8 ± 63.1 [218.8, 236.7]	90.2 ± 76.0 [76.2, 104.1]
Reference vessel diameter (visual estimate) (mm)	4.7 ± 1.1 [4.6, 4.7]	5.0 ± 0.8 [5.0, 5.1]	4.6 ± 1.0 [4.5, 4.8]	5.0 ± 0.8 [4.9, 5.2]
Diameter stenosis (visual estimate) (%)	86.9 ± 12.8 [86.2, 87.7]	87.0 ± 12.7 [86.0, 87.9]	93.3 ± 10.6 [91.7, 94.8]	81.9 ± 13.4 [79.4; 84.3]
Calcification				
None	23.9% (259/1082)	20.7% (143/691)	15.1% (29/192)	43.1% (50/116)
Mild	31.4% (340/1082)	32.7% (226/691)	33.3% (64/192)	29.3% (34/116)
Moderate	29.2% (316/1082)	29.8% (206/691)	29.2% (56/192)	12.9% (15/116)
Heavy	15.4% (167/1082)	16.8% (116/691)	22.4% (43/192)	14.7% (17/116)
TASC classification				
A	38.1% (408/1071)	36.5% (252/690)	3.1% (6/192)	37.4% (43/115)
B	29.3% (314/1071)	34.1% (235/690)	15.1% (29/192)	41.7% (48/115)
C	18.6% (199/1071)	18.4% (127/690)	41.7% (80/192)	14.8% (17/115)
D	14.0% (150/1071)	11.0% (76/690)	40.1% (77/192)	6.1% (7/115)
Mean lesions per subjects	1.2 ± 0.5 [1.2, 1.3]	1.2 ± 0.5 [1.17, 1.24]	1.1 ± 0.4 [1.1, 1.2]	1.3 ± 0.6 [1.2, 1.4]
Target lesion location				
Common femoral	1.0% (11/1084)	0%	0% (0/192)	0% (0/116)
Superficial femoral artery	54.3% (589/1084)	85.2% (589/691)	69.3% (133/192)	73.3% (85/116)
Popliteal artery	21.1% (229/1084)	14.8% (102/691)	7.3% (14/192)	12.9% (15/116)
Anterior tibial artery	5.8% (63/1084)	0%	6.8% (13/192)	0% (0/116)
Posterior tibial artery	4.2% (46/1084)	0%	3.6% (7/192)	0.9% (1/116)
Tibioperoneal trunk	3.7% (40/1084)	0%	0% (0/192)	1.7% (2/116)
Peroneal artery	3.3% (36/1084)	0%	2.1% (4/192)	0.9% (1/116)
Others^b^	6.5% (70/1084)	0%	10.9% (21/192)	10.3% (12/116)

Continuous data are presented as the means ± standard deviation and [95%CI]; categorical data are given as the n/N (percentage)

^a^ Superficial femoral artery and proximal popliteal artery

^b^ bypass, iliac artery, diffuse lesions extending in more than one artery, profunda femoris, dorsalis pedis

Vessel preparation was performed in 73.1% of cases, predominantly with a plain balloon (88.4% of the pre-treated lesions), but also with cutting or scoring balloons (6.4%) and atherectomy devices (3.7%) (Table 3). Additional stenting was required in 15.7% of lesions (170/1084).

**Table 3:** Procedural characteristics

Description	Full cohort *N* = 1084	Femoropopliteal lesions ^a^ *N* = 691	Long lesions (≥ 15 cm) *N* = 192	In-stent restenosis *N* = 116
Target lesion preparation (multiple answers possible)	73.1% (792/1084)	71.3% (493/691)	85.9% (165/192)	58.6% (68/116)
Plain old balloon	88.4% (700/792)	88.6% (437/493)	92.1% (152/165)	89.7% (61/68)
Rotational thrombectomy	3.5% (28/792)	3.2% (16/493)	5.5% (9/165)	0% (0/68)
Scoring balloon	3.0% (24/792)	3.7% (18/493)	1.8% (3/165)	2.9% (2/68)
Cutting balloon	3.4% (27/792)	4.7% (23/493)	3.0% (5/165)	5.9% (4/68)
Atherectomy device	3.7% (29/792)	2.0% (10/493)	2.4% (4/165)	1.5% (1/68)
Drug-coated balloon*	0.9% (7/792)	1.2% (6/493)	3.0% (5/165)	0% (0/68)
Stent	0.3% (2/792)	0.2% (1/493)	0% (0/165)	2.5% (1/68
Other	1.5% (12/792)	1.8% (9/493)	3.0% (5/165)	0% (0/68)
Bailout stenting	15.7% (170/1084)	20.3% (140/691)	27.1% (52/192)	7.8% (9/116)
Device success^b^	99.8% (1448/1451)	100% (939/939)	100% (445/445)	100% (154/154)
Technical success^c^	98.4% (1067/1084)	98.4% (680/691)	98.4% (189/192)	98.3% (114/116)
Procedural success^d^	96.6% (847/1084)	97.1% (575/592)	96.8% (181/187)	97.1% (100/103)

Continuous data are presented as the means ± standard deviation and [95%CI]; categorical data are given as the counts (percentage)

^a^Superficial femoral artery and proximal popliteal artery

^b^ Successful delivery, inflation, deflation and retrieval of Passeo-18 lux

^c^Successful completion of the endovascular procedure and immediate morphological success with ≤ 50% residual diameter reduction of the treated lesion as determined by visual estimation

^d^Technical and device success without the occurrence of any major adverse events during the hospital stay

* Lesion has been treated with Passeo-18 lux and a DCB from a different manufacturer

**Fig. 1:**
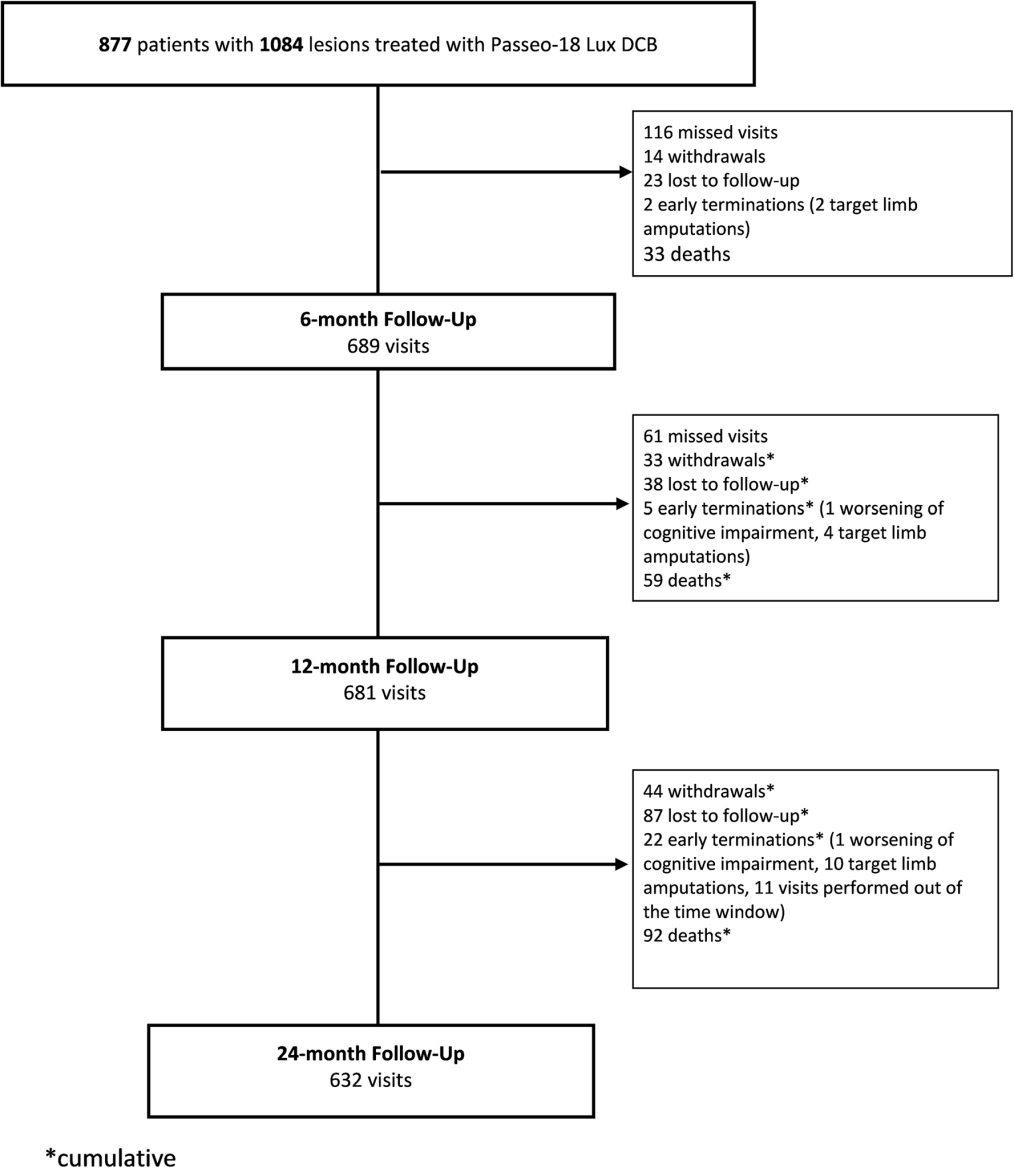
Subjects disposition

**Fig. 2:**
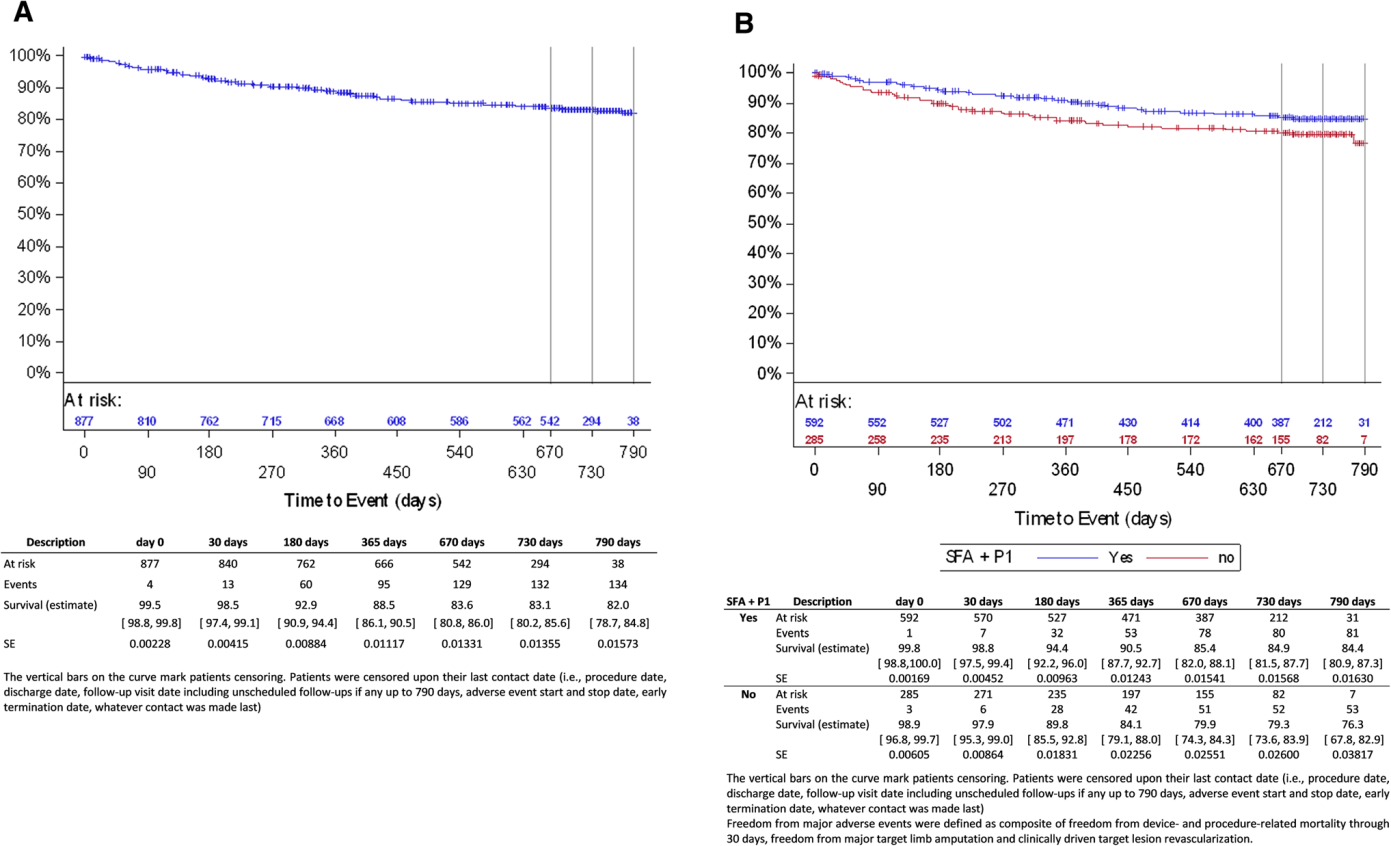
**A** Kaplan–Meier curve for freedom from major adverse events (full cohort). **B** Kaplan–Meier curve for freedom from major adverse events (femoropopliteal subgroup)

**Fig. 3:**
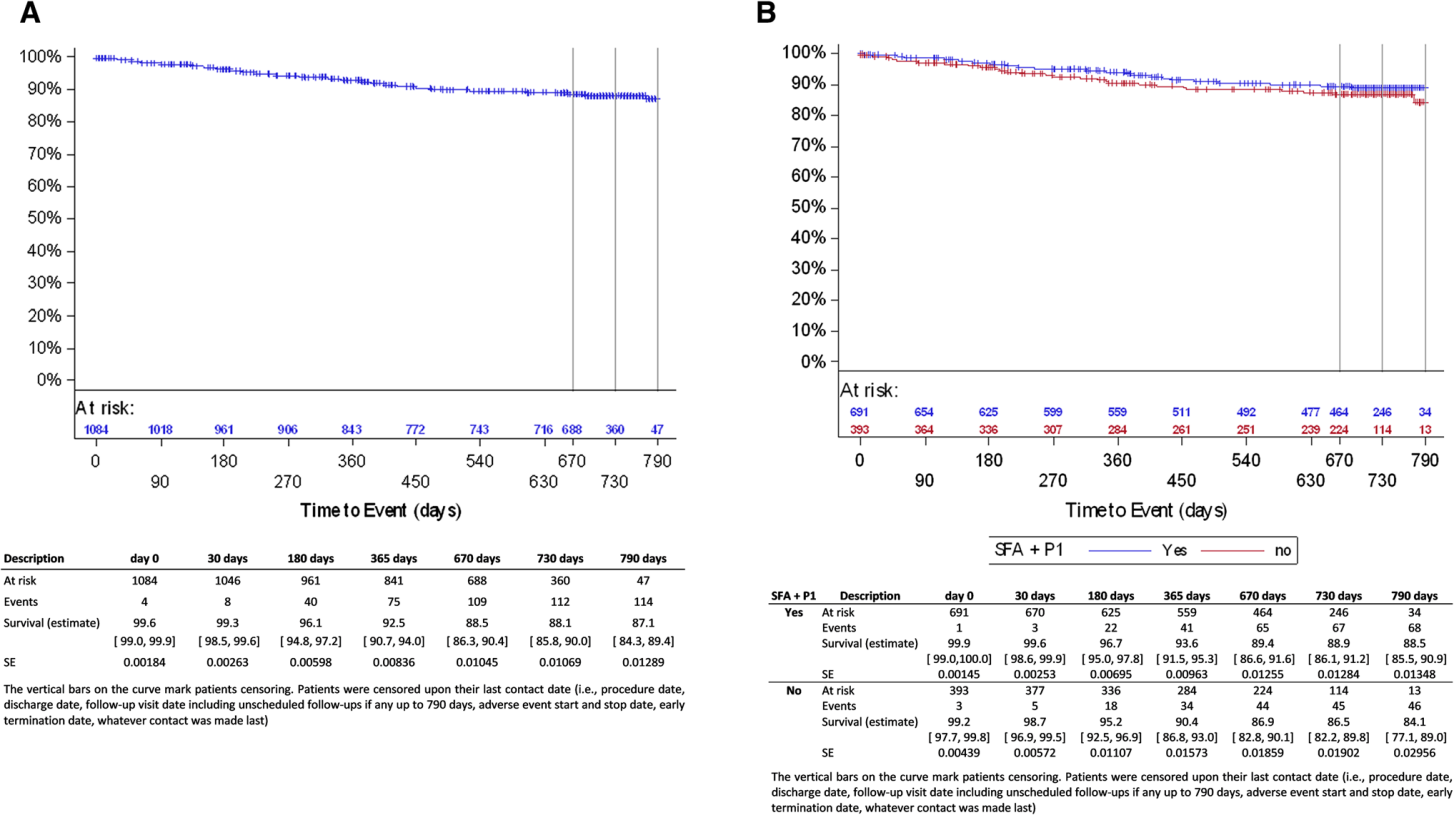
**A** Kaplan–Meier curve for freedom from clinically driven target lesion revascularization (full cohort, lesion based). **B** Kaplan–Meier curve for freedom from clinically driven target lesion revascularization (femoropopliteal subgroup, lesion based)

Patient disposition at follow-up in the full cohort is displayed in Fig. 1. The primary safety endpoint, freedom from MAE, was 92.9% (95% CI [90.9, 94.4]) at 6 months, 88.5% (95% CI [86.1, 90.5]) at 12 months and 83.1% (95% CI [80.2, 85.6]) at 24 months (Fig. 2A, Table 4). At 12 and 24 months, the primary performance endpoint, freedom from clinically driven TLR, was 92.5% (95% CI [90.7, 94.0]) and 88.1% (95% CI [85.8, 90.0]), respectively (Fig. 3A, Table 4). At 24 months, 29 subjects had undergone a major target limb amputation of which 24 were diabetics and 13 had Rutherford class 5 or higher at baseline. All amputations were adjudicated by the CEC as not device related. Seven patients died within 30 days after the index procedure. Through the 24-month follow-up, the all-cause mortality by Kaplan–Meier estimate was 12.0% (95% CI [9.9, 14.6]). Up to 790 days, 94 patients died of which 46 were Rutherford 5 or 6 (Supplemental online Fig. 1A). None of the deaths which causes are listed in supplemental online Table 2 was considered as procedure- or device related by the CEC.

**Table 4:** Kaplan–Meier analyses of clinical and performance outcomes through subgroups

	Full cohort		Femoropopliteal ^a^		Long lesions (≥ 15 cm)		In-stent restenosis	
# Subjects/lesion	877subjects/1084 lesions (Imaging cohort: 289 subjects/345 lesions)		592subjects/691 lesions (Imaging cohort: 205 subjects/233 lesions)		187subjects/192 lesions (Imaging cohort: 75 subjects/75 lesions)		103subjects/116 lesions (Imaging cohort: 48 subjects/ 54 lesions)	
Description	365 days	730 days	365 days	730 days	365 days	730 days	365 days	730 days
Major adverse events^b^	11.5% [9.5, 13.9]	16.9% [14.4, 19.8]	9.5% [7.3, 12.3]	15.1% [12.3, 18.5]	14.1% [9.7, 20.1]	22.3% [16.7, 29.6]	12.2% [7.1, 20.5]	27.5% [19.4, 38.1]
Clinically driven Target lesion revascularization^c^	7.5% [6.0, 9.3]	11.9% [10.0, 14.2]	6.4% [4.7, 8.5]	11.1% [8.8, 13.9]	12.2% [8.2, 17.9]	19.7% [14.4, 26.6]	10.8% [6.3, 18.3]	21.6% [14.7, 31.0]
Amputation (target limb)	6.0% [4.6, 7.8]	7.3% [5.7, 9.3]	3.6% [2.3, 5.5]	4.4% [3.0, 6.5]	5.8% [3.2, 10.5]	7.2% [4.1, 12.3]	3.2% [1.0, 9.6]	4.5% [1.7, 11.6]
Major amputation (target limb)	3.1% [2.1, 4.5]	3.5% [2.4, 5.0]	2.0% [1.1, 3.6]	2.4% [1.4, 4.2]	2.9% [1.2, 6.9]	3.6% [1.6, 7.9]	0.0% [0.0, 0.0]	0.0% [0.0, 0.0]
Amputation free survival^d^	91.2% [89.1, 92.9]	85.8% [83.1, 88.1]	92.3% [89.8, 94.3]	88.2% [85.1, 90.7]	91.3.% [86.0, 94.7]	84.0% [77.3, 88.9]	92.4% [84.7, 96.3]	87.0% [77.4, 92.7]
Death	6.6% [5.1, 8.5]	12.0% [9.9, 14.6]	6.2% [4.5, 8.5]	10.2% [7.9, 13.2]	7.2% [4.2, 12.1]	14.8% [10.2, 21.2]	7.0% [3.4, 14.1]	12.0% [6.7, 20.9]
Primary patency^e^	84.5% [82.1, 86.6]	75.9% [72.9, 78.7]	84.6% [81.6, 87.2]	75.9% [72.1, 79.2]	74.5% [67.4, 80.3]	61.8% [53.5, 69.1]	77.3% [68.3, 84.1]	58.5% [47.7, 67.8]
Primary patency (imaging cohort)^f^	79.1% [74.4, 83.1]	65.6% [60.2, 70.5]	81.1% [75.5, 85.6]	67.2% [60.6, 73.0]	73.3% [61.8, 81.9]	58.6% [45.9, 69.2]	77.8% [64.2, 86.7]	55.5% [40.5, 68.1]

Data are presented as Kaplan–Meier estimates and [95%CI)

^a^Superficial femoral artery and proximal popliteal artery

^b^ Major adverse events are defined as composite of device- and procedure-related mortality through 30 days, and major target limb amputation and clinically driven target lesion revascularization, ^c^ per lesion

^d^ A composite of death and target limb major amputation

^e^ Defined as freedom from > 50% restenosis in the target lesion as indicated by a duplex ultrasound peak systolic velocity ratio (PSVR) > 2.5 or by visual assessment of an angiogram with no clinically driven re-intervention. Note: Duplex ultrasound was not mandated by the protocol and was not available in all patients

^f^All subjects who have had either a duplex ultrasound or an angiogram at the 12 or 24-month visit.

The Kaplan–Meier estimate of secondary endpoint, primary patency, was 84.5% (95% CI [82.1, 86.6]) at 12 months and 75.9% (95% CI [72.9, 78.7]) at 24 months (Supplemental online Fig. 2A, Table 4). However, inherent to an observational study, duplex ultrasound assessment was not mandatory. Therefore, non-symptomatic binary restenosis has not been determined for all patients. In the subgroup of patients with imaging assessment done (289 subjects/345 lesions), the patency rates were 79.1% (95% CI [74.4, 83.1]) and 65.6% (95% CI [60.2, 70.5]) at 12 and 24 months (Table 4). Using paired data, ABI improved significantly by 0.2 from baseline to 12- and 24-month follow-up, and 81.7% improved of at least 1 Rutherford class between baseline and 24-month follow-up (Supplemental online Table 1).

### Femoropopliteal Lesions, Long Lesions and In-Stent Restenosis Sub-analysis

Three subgroups analysis were performed: patients with at least one target lesion in the superficial femoral artery (SFA) and or the proximal popliteal artery (592 patients, 691 lesions), long lesions (187 patients, 192 lesions) and patients with ISR (103 patients, 116 lesions).

Baseline demographics, lesion characteristics and procedure detail are presented in Tables 1, 2 and 3.

In the femoropopliteal population, freedom from MAE was 94.4% (95% CI [92.2, 96.0]) at 6 months, 90.5% (95% CI [87.7, 92.7]) at 12 months and 84.9% (95% CI [81.5, 87.7]) at 24 months (Fig. 2B, Table 4). At 12 and 24 months, freedom from clinically driven TLR was 93.6% (95% CI [91.5, 95.3]) and 88.9% (95% CI [86.1, 91.2]), respectively (Fig. 3B, Table 4). The patency rates were 84.6% (95% CI [81.6, 87.2]) at 12 months and 75.9% (95% CI [72.1, 79.2]) at 24 months, 81.1% (95% CI [75.6, 85.6]) and 67.2% (95% CI [60.6, 73.0]) in the corresponding imaging cohort (205 subjects/233 lesions, Table 4).

The all-cause mortality rate was 6.2% (95% CI [4.5, 8.5]) at 12 months and 10.2% (95% CI [7.9, 13.2]) at 24 months. At 790 days, 54 patients had died of which 32 were Rutherford 5 or 6 (Supplemental online Fig. 1B, Table 4). Excluding patients with CLI from the femoropopliteal subgroup, 12- and 24-month mortality rates were 3.0% (95% CI [1.6, 5.5]) and 5.4% (95% CI [3.4, 8.6]), with 19 deaths at 790 days.

Twelve- and 24-month freedom from MAE was 85.9% (95% CI [79.9, 90.3] and 77.7% (95% CI [70.4, 83.3] in the long lesions subgroup; 87.8% (95% CI [79.5, 92.9]) and 72.5% (95% CI [61.9, 80.6]) in the ISR subgroup. Freedom from clinically driven TLR at 12 and 24 months was 87.8% (95% CI [82.1, 91.8]) and 80.3% (95% CI [73.4, 85.6]) in the long lesions subgroup, 89.2% (95% CI [81.7, 93.7]) and 78.4% (95% CI [69.0, 85.3]) in the ISR subgroup.

All-cause mortality was 7.2% (95% CI [4.2, 12.1]) at 12 months and 14.8% (95% CI [10.2, 21.2]) (25 deaths at 790 days) at 24 months in the long lesions subgroup. In the ISR subgroup, all-cause of death was 7.0% (95% CI [3.4, 14.1]) at 12 months and 12.0% (95% CI [6.7, 20.9]) at 24 months (11 deaths at 790 days).

### Multivariate Analysis

The multivariate Cox regression analysis performed to identify predictors of mortality in the study population showed that age, presence of diabetes, renal disease and CLI are among the main covariates that are predictors of death, while dose of paclitaxel administered during the index procedure is not (*p* value 0.8627) (Table 5).

**Table 5:** Cox regression: mortality in BIOLUX P-III up to 730 days

Parameter	Hazard ratio (95% CI)	*p* Value
Paclitaxel dose (mg)	1.007 [0.929; 1.091]	0.8627
Age (≥ 75 years vs. < 75 years)	2.486 [1.557; 3.968]	0.0001
Diabetes	1.836 [1.137; 2.965]	0.0130
Renal disease	2.389 [1.482, 3.849]	0.0003
CLI	2.781 [1.693; 4.569]	< 0.0001
Cancer	1.705 [0.920; 3.162]	0.0903

## Discussion

The BIOLUX P-III all-comers single-arm study was designed to further assess Passeo-18 lx DCB safety and clinical performance in a broad population. The full cohort reflects a real-world population without any lesion, procedural or relevant patient restrictions. As a result, the cohort encompasses a higher risk patient population with 47.7% being diabetics and 42.1% CLI (13.2% in Rutherford class 4, 21.6% in Rutherford class 5, 7.3% in Rutherford class 6) compared to the pivotal randomized studies [2–7], but also contemporary global DCB registries [14–17]. As patients with CLI and below-the-knee lesions represent a different and more severe pattern of the peripheral artery disease, further reports of the BIOLUX P-III study will present outcomes in these subgroups.

The results from the BIOLUX P-III study in the full cohort and across subgroups are in line with the previous BIOLUX P-I study and show continued safety and efficacy of Passeo-18 lx DCB in infrainguinal artery. At 24 months, the clinically driven TLR rate in the full cohort was 11.9%. These outcomes are comparable to published DCB registries which enrolled less challenging patients, excluding below-the-knee lesions as well as patients with Rutherford 5 or 6 [14–17]. The MAE rates, 11.5% and 16.9% at, respectively, 12 and 24 months in the full cohort were predominantly due to clinically driven TLR. At 24 months, the major target limb amputation rates, 3.5% in the full cohort and 2.4% in the femoropopliteal lesions subgroup, were a bit higher than in other global DCB registries [14–17]. This difference is likely attributed to the significant percentage of diabetic and CLI patients, with more patients with minor and severe tissue loss at baseline in BIOLUX P-III.

All-cause of death by Kaplan–Meier estimates was 6.6% and 12.0% at 12 and 24 months in the full cohort. These numbers are above the mortality rates reported in similar DCB registries [14–16], which are between 0.6% and 3.5% at 1 year, and between 5.9% and 7% at 2 years. However, in the non-CLI femoropopliteal population of our data set, the mortality rates, 3.0% and 5.4% at, respectively, 12 and 24 months, are comparable to published data. A recent study level meta-analysis [18] of randomized controlled trials with paclitaxel-coated devices (drug-coated balloon and drug-eluting stent) used for the treatment of atherosclerotic femoropopliteal artery raised an important concern about an increase in patient mortality from 2 years after treatment with drug-coated devices compared to non-drug-coated devices. The VIVA physician’s performed an independent patient data (IPD) meta-analysis [19] which confirmed an increased long-term mortality risk with paclitaxel devices, but with a much smaller signal. It should also be noted that the RCTs included in these meta-analyses were not powered to investigate long-term mortality between the study arms. In contrast, large health insurance database analysis showed no increased in all-cause mortality with paclitaxel devices. In an analysis of more than 16,000 patients in the USA, including 51% with CLI, the cumulative incidence of all-cause mortality after femoropopliteal artery revascularization was 32.5% with paclitaxel-coated devices versus 34.3% for patient treated with non-drug-coated devices through 600 days post-procedure. Among patients with CLI, the cumulative incidence of mortality through 600 days post-procedure was 38.1% with drug-coated devices versus 40.1% for non-drug-coated devices [20]. Similarly, the analysis of more than 20,000 patients from the German BARMER Health Insurance database, treated with paclitaxel devices, reported no increased mortality in patients receiving paclitaxel-coated devices compared to those receiving balloon angioplasty or bare metal stents over 11-year follow-up [21]. In BIOLUX P-III, the multivariate analysis performed on the full cohort showed no relationship between paclitaxel dose and an increase in mortality at 24 months. Besides, it has to be noted that unlike what was reported in the meta-analysis [18], in the full cohort, we observed a slight decrease in mortality during the second year compared to the first year.

The follow-up compliance in BIOLUX P-III was lower than anticipated. In addition to the observational study design, an explanatory hypothesis is that the follow-up compliance is lower when a treatment is associated with a better efficacy. Besides, patients experiencing an improvement in their symptoms are less likely to adhere to study-specific follow-up visits. While major adverse events such as revascularization or major amputation that require the patients to return to the site for treatment are unlikely to be underreported, the vital status of patients lost to follow-up is missing. Therefore, BIOLUX P-III has been extended to 5-year follow-up and steps have been taken to obtain vital status data on patients who were lost to follow-up or withdrew.

## Limitation

Limitations of BIOLUX P-III are those inherent to a single-arm study such as lack of randomization which limits the comparability to other devices. Registries also have the possibility of underreporting; however, the selected clinical endpoints are prominent events reducing the risk that they were overlooked by the sites. Furthermore, all MAEs, death and TLR were adjudicated by a CEC. A major limitation was that freedom from > 50% restenosis could not be systematically assessed as by default, observational studies allow only treatment according to standard of care. Therefore, the patency rate in the non-predefined imaging cohort, i.e., only patients with imaging assessment performed at 12 months or 24 months, are also reported. Likewise, performance outcomes such as ABI or Rutherford class measurement or questionnaires were not available for all patients. Lastly, the general follow-up compliance was not optimal.

## Conclusion

To our knowledge, BIOLUX P-III is the only large DCB single-arm study to report outcomes in a real-world population including high rates of diabetics as well as Rutherford 5 and 6 patients. The 24-month results in the full cohort and the subgroups confirm Passeo-18 lx DCB safety and clinical performance in a large patient population with infrainguinal lesions treated under real-world condition. The outcomes were good and comparable to DCB registries investigating femoropopliteal arteries. With regard to the mortality signal raised in a recent meta-analysis, a 5‐year follow‐up time point is being added to the study in order to further investigate a potential relationship between mortality and paclitaxel.

## Electronic supplementary material

Below is the link to the electronic supplementary material.

Supplementary file1 (DOCX 44 kb)

Supplementary file2 (TIF 1334 kb)

Supplementary file3 (TIF 1677 kb)

Supplementary file4 (TIF 1407 kb)

Supplementary file5 (TIF 1772 kb)
