# Correction to: Paclitaxel-Coated Balloon Angioplasty for the Treatment of Infrainguinal Arteries: 24-Month Outcomes in the Full Cohort of BIOLUX P-III Global Registry

**DOI:** 10.1007/s00270-021-02862-w

**Published:** 2021-05-07

**Authors:** Gunnar Tepe, Thomas Zeller, Matej Moscovic, Jean-Marc Corpataux, Johnny Kent Christensen, Koen Keirse, Giovanni Nano, Henrik Schroeder, Christoph A. Binkert, Marianne Brodmann

**Affiliations:** 1 Department of Radiology, Klinikum Rosenheim, Rosenheim, Germany; 2 Clinic Cardiology and Angiology II, Universitäts-Herzzentrum Freiburg – Bad Krozingen, Bad Krozingen, Germany; 3 Angiology Clinic, Institute of Cardiovascular Diseases, Kosice, Slovakia; 4 Department of Vascular Surgery, Lausanne University Hospital, Lausanne, Switzerland; 5 Department of Radiology, Kolding Hospital, Kolding, Denmark; 6 Department of Vascular Surgery, Regional Hospital Heilig Hart, Tienen, Belgium; 7 1st Vascular Surgery Department, IRCCS Policlinico San Donato, San Donato Milanese, Italy; 8 Center for Diagnostic Radiology and Minimally Invasive Therapy, Jewish Hospital, Berlin, Germany; 9 Radiology Institute, Kantonsspital Winterthur, Winterthur, Switzerland; 10 Division of Angiology, Department of Internal Medicine, Medical University Graz, Graz, Austria; 11 Institut für Diagnostische Und Interventionelle Radiologie, RoMed Klinikum Rosenheim, Pettenkoferstr. 10, 83022 Rosenheim, Germany

## Correction to: Cardiovasc Intervent Radiol (2021) 44:207–217 10.1007/s00270-020-02663-7

The original version of this paper did not contain a list of BIOLUX P-III investigators. The purpose of this addendum is to acknowledge the contribution of all investigators who participated in the study.

**Collaborators: BIOLUX P-III Global Registry Investigators:** Marianne Brodmann, Thomas Zeller, Jean-Marc Corpataux, Matej Moscovic, Gunnar Tepe, Koen Keirse, Giovanni Nano, Johannes B. Dahm, Johnny Kent Christensen, Reza Ghotbi, Christoph Binkert, Henrik Schröder, Denis Henroteaux, John Wang Chaw Chian, Eric Rosset, Enrique Alejandre Lafont, Sabrina Houthoofd, Miguel Araujo; Shaiful Azmi Yahaya, Don Robertson, Martin Freund, Lonneke Yo, Uei Pua, Roxanne Wu, Frank Hammer, Michael Lichtenberg, Janne Korhonen, Della Schiava, Ralf Langhoff, Stefano Michelagnoli, Secundino Llagostera, Jose-Maria Romero, Max Amor, Daniel Kretzschmar, Steven Kum, Patrice Mwipatayi, Albert J Smeets, Francisco Javier Serrano Hernando, Jost Philipp Schäfer, Gil Marques, Jos C. van den Berg, Ramesh Velu, Karlis Kupcs

