# Supplementary material for: Paclitaxel-Coated Balloon Angioplasty for the Treatment of Infrainguinal Arteries: 24-Month Outcomes in the Full Cohort of BIOLUX P-III Global Registry
Source: Cardiovasc Intervent Radiol. 2020 Oct 20;44(2):207–17. doi: 10.1007/s00270-020-02663-7 (PMC7806550; doi:10.1007/s00270-020-02663-7)
Supplement: Supplementary file 1 — Supplementary file1 (DOCX 44 kb) [file 270_2020_2663_MOESM1_ESM.docx]

**Table 1 Hemodynamic outcome and clinical success at baseline and follow-up**

|  | **Full cohort** | | | | **Femoropopliteal ^(1)^** | | | | **Long lesions (≥15cm)** | | | | **In stent restenosis** | | | |
| --- | --- | --- | --- | --- | --- | --- | --- | --- | --- | --- | --- | --- | --- | --- | --- | --- |
| **Mean ABI (target limb)** | **Baseline**  **N=430** | **12 months**  **N= 304** | | **24 months**  **N=232** | **Baseline**  **N=318** | **12 months**  **N= 230** | | **24 months**  **N=171** | **Baseline**  **N=96** | **12 months**  **N= 76** | | **24 months**  **N=61** | **Baseline**  **N=63** | **12 months**  **N= 48** | | **24 months**  **N=40** |
| Mean ± SD  [95% CI] | 0.66 ± 0.23  [0.64, 0.68] | 0.86 ± 0.20  [0.84, 0.89] | | 0.86 ± 0.22  [0.84, 0.89] | 0.64 ± 0.22  [0.62, 0.67] | 0.88 ± 0.19  [0.85, 0.90] | | 0.87 ± 0.20  [0.84, 0.90] | 0.59 ± 0.19  [0.55, 0.63] | 0.80 ± 0.22  [0.75, 0.86] | | 0.80 ± 0.19  [0.75, 0.85] | 0.66 ± 0.20  [0.61, 0.72] | 0.78 ± 0.21  [0.72, 0.84] | | 0.90 ± 0.18  [0.84, 0.96] |
| **Change in ABI compared to baseline**  **(paired data)** | **12 months**  **N=207** | | **24 months**  **N=164** | | **12 months**  **N=161** | | **24 months**  **N=123** | | **12 months**  **N=55** | | **24 months**  **N=48** | | **12 months**  **N=33** | | **24 months**  **N=31** | |
| Mean ± SD  Min - Max  Signed Rank | 0.21 ± 0.25  -0.6 - 1.03  <.0001 | | 0.21 ± 0.27-  -0.6 - 1.03  <.0001 | | 0.22 ± 0.24  -0.6 - 1.03  <.0001 | | 0.20 ± 0.26  -0.6 - 1.03  <.0001 | | 0.24 ± 0.22  -0.25 - 0.61  <.0001 | | 0.19 ± 0.25  -0.6 - 0.7  <.0001 | | 0.11 ± 0.24  -0.6 - 0.69  0.0045 | | 0.21 ± 0.23  -0.2 - 0.82  <.0001 | |
| **Improvement in Rutherford compared to baseline**  **(paired data)** | **12 months**  **N=460** | | **24 months**  **N=405** | | **12 months**  **N=327** | | **24 months**  **N=291** | | **12 months**  **N=115** | | **24 months**  **N=104** | | **12 months**  **N=58** | | **24 months**  **N=50** | |
| Improved  Equal  Worsened | 81.7% (376)  14.8% (68)  3.5% (16) | | 81.7% (331)  15.3% (62)  3.0% (12) | | 81.7% (267)  15.0% (49)  3.4% (11) | | 81.8% (238)  15.8% (46)  2.4% (7) | | 83.5% (96)  14.8% (17)  1.7% (2) | | 78.8% (82)  20.2% (21)  1.0% (1) | | 81.0% (47)  12.1% (7)  6.9% (4) | | 78.0% (39)  16.0% (8)  6.0% (3) | |

Continuous data are presented as the means ± standard deviation, Min – Max and [95%CI]; categorical data are given as the counts (percentage). ABI: ankle brachial index, PAD-peripheral artery disease. (1) Superficial femoral artery and proximal popliteal artery.

**Table 2: Causes of Death**

| **Category of death** | **Deaths Full cohort, n** | **Diabetes, n (%)** | **CLI, n (%)** | **BTK lesion, n (%)** |
| --- | --- | --- | --- | --- |
| Cancer | 14 | 9 (64.3) | 7 (50.0) | 3 (21.4) |
| Car accident | 1 | 0 (0.0) | 1 (100.0) | 0 (0.0) |
| Cardiac death | 29 | 20 (69.0) | 11 (37.9) | 10 (34.5) |
| Cerebral trauma | 1 | 0 (0.0) | 0 (0.0) | 0 (0.0) |
| Gastrointestinal bleeding | 1 | 0 (0.0) | 0 (0.0) | 0 (0.0) |
| Gastrointestinal ischemia | 4 | 1 (25.0) | 3 (75.0) | 2 (50.0) |
| Infection | 13 | 9 (69.2) | 10 (76.9) | 3 (23.1) |
| Multiorgan failure | 1 | 0 (0.0) | 1 (100.0) | 0 (0.0) |
| Progression of PAD | 4 | 4 (100.0) | 3 (75.0) | 2 (50.0) |
| Pulmonary embolism | 1 | 1 (100.0) | 1 (100.0) | 1 (100.0) |
| Renal failure | 3 | 3 (100.0) | 3 (100.0) | 0 (0.0) |
| Respiratory failure | 2 | 1 (50.0) | 1 (50.0) | 1 (50.0) |
| Stroke | 4 | 1 (25.0) | 3 (75.0) | 1 (25.0) |
| Unknown* | 16 | 11 (68.8) | 11 (68.8) | 3 (18.8) |
| Total | 94 | 60 (63.8) | 55 (58.5) | 26 (27.7) |

* 6 patients died at home with no further information available

PAD : peripheral artery disease

**Table 3: Enrolment per site, country and continent**

| **Continent** | **Country** | **Site** | **# Subjects** |
| --- | --- | --- | --- |
| Asia | Malaysia | MYS001 | 13 |
|  | Singapore | SGP001 | 12 |
|  |  | SGP002 | 4 |
|  |  | SGP003 | 20 |
| Australia | Australia | AUS001 | 13 |
|  |  | AUS002 | 11 |
|  |  | AUS003 | 4 |
|  |  | AUS004 | 2 |
| Europe | Austria | AUT001 | 146 |
|  |  | AUT002 | 13 |
|  | Belgium | BEL001 | 14 |
|  |  | BEL002 | 21 |
|  |  | BEL003 | 37 |
|  |  | BEL004 | 10 |
|  | Denmark | DNK001 | 29 |
|  | Finland | FIN001 | 9 |
|  | France | FRA001 | 17 |
|  |  | FRA002 | 5 |
|  |  | FRA003 | 7 |
|  |  | FRA004 | 8 |
|  | Germany | GER001 | 93 |
|  |  | GER002 | 6 |
|  |  | GER003 | 35 |
|  |  | GER004 | 3 |
|  |  | GER005 | 41 |
|  |  | GER006 | 8 |
|  |  | GER007 | 10 |
|  |  | GER008 | 15 |
|  |  | GER009 | 23 |
|  |  | GER010 | 24 |
|  | Italy | ITA001 | 37 |
|  |  | ITA002 | 8 |
|  | Latvia | LVA001 | 1 |
|  | Netherlands | NLD001 | 13 |
|  |  | NLD002 | 4 |
|  | Portugal | PRT001 | 3 |
|  | Slovakia | SVK001 | 48 |
|  | Spain | ESP001 | 4 |
|  |  | ESP002 | 8 |
|  |  | ESP003 | 8 |
|  |  | ESP004 | 14 |
|  | Switzerland | CHE001 | 3 |
|  |  | CHE002 | 49 |
|  |  | CHE003 | 24 |
