# Supplementary figures and images for: Paclitaxel-Coated Balloon Angioplasty for the Treatment of Infrainguinal Arteries: 24-Month Outcomes in the Full Cohort of BIOLUX P-III Global Registry
Source: Cardiovasc Intervent Radiol. 2020 Oct 20;44(2):207–17. doi: 10.1007/s00270-020-02663-7 (PMC7806550; doi:10.1007/s00270-020-02663-7)

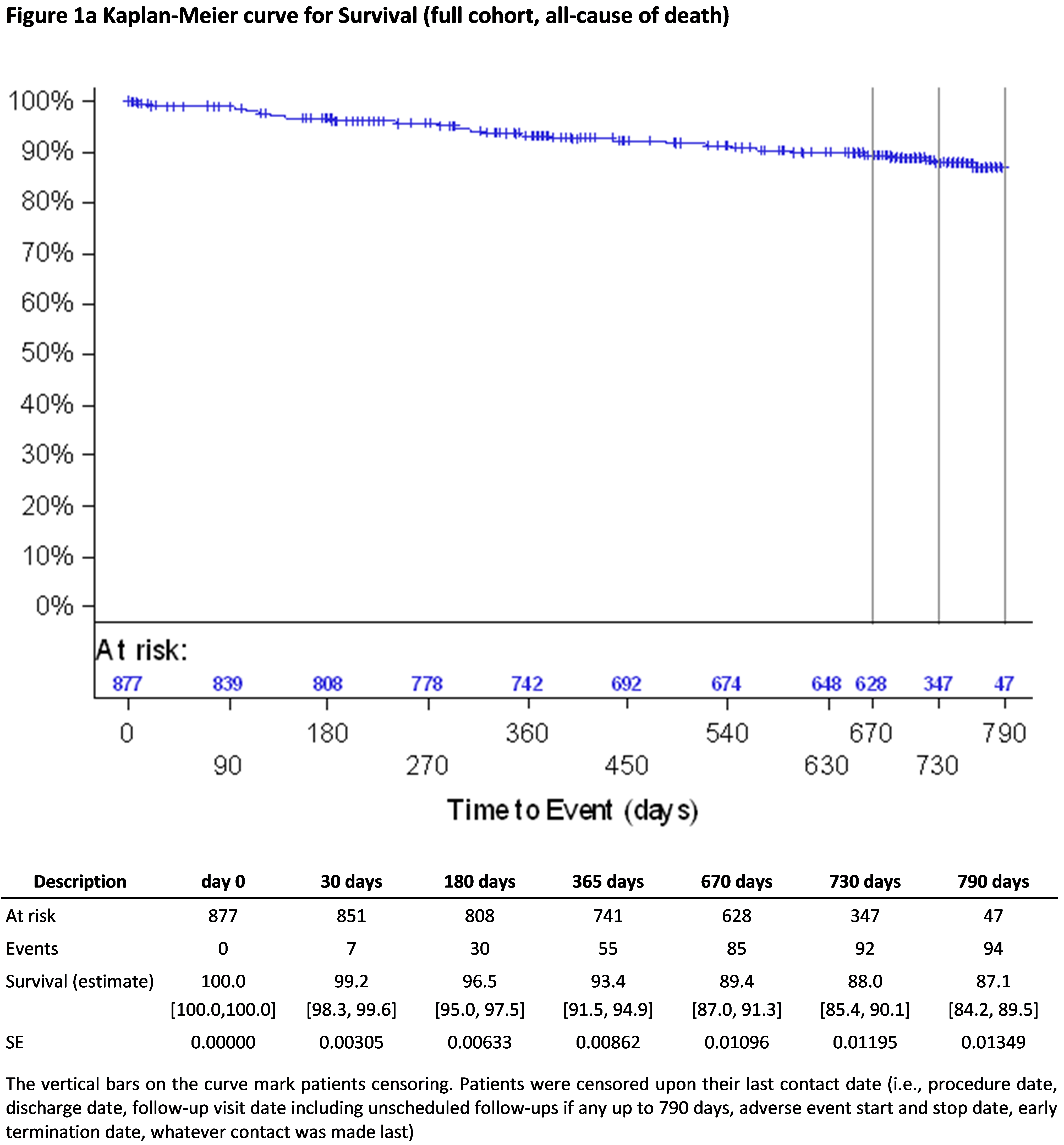

Supplement: Supplementary file 2 — Supplementary file2 (TIF 1334 kb) [file 270_2020_2663_MOESM2_ESM.tif]

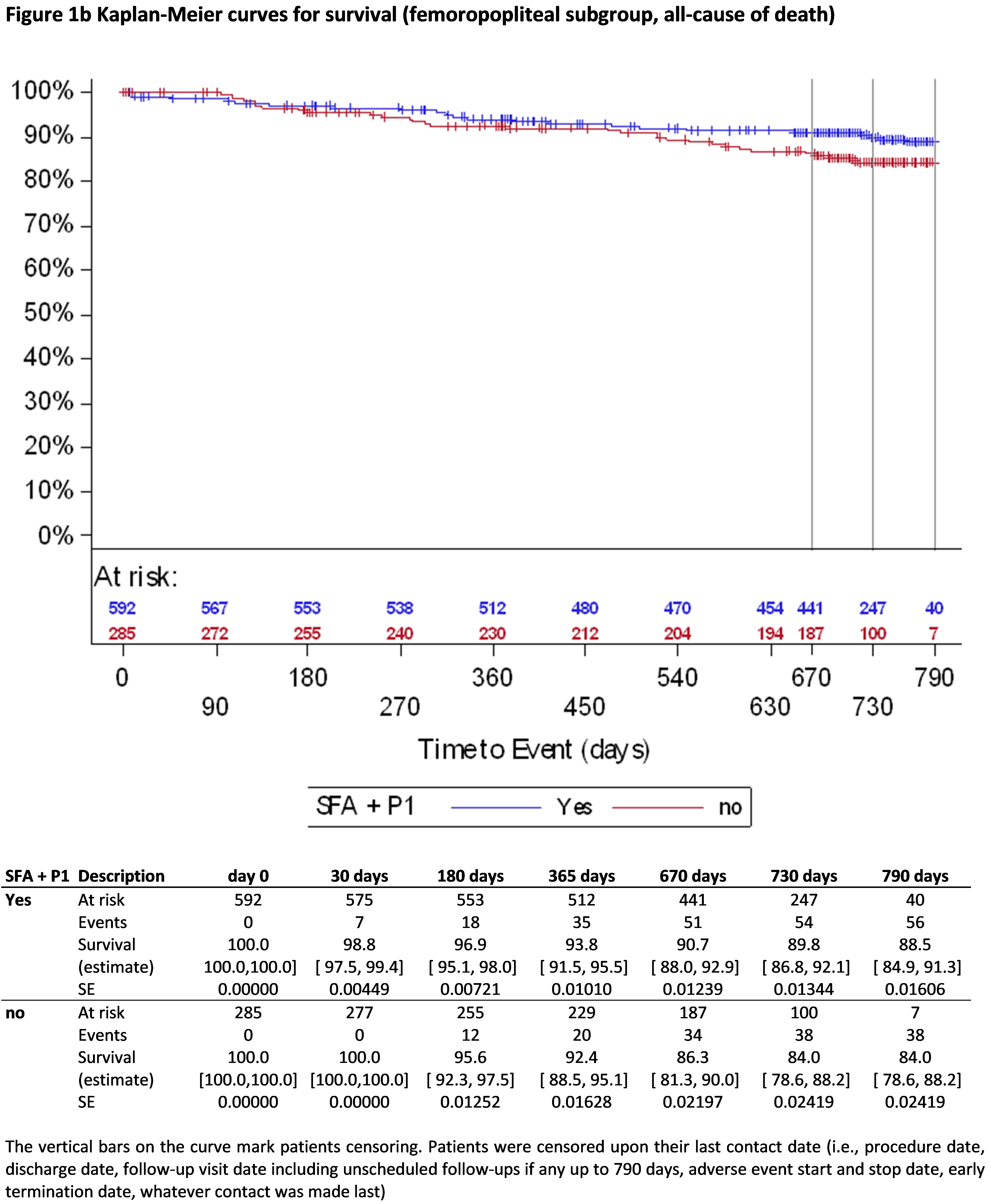

Supplement: Supplementary file 3 — Supplementary file3 (TIF 1677 kb) [file 270_2020_2663_MOESM3_ESM.tif]

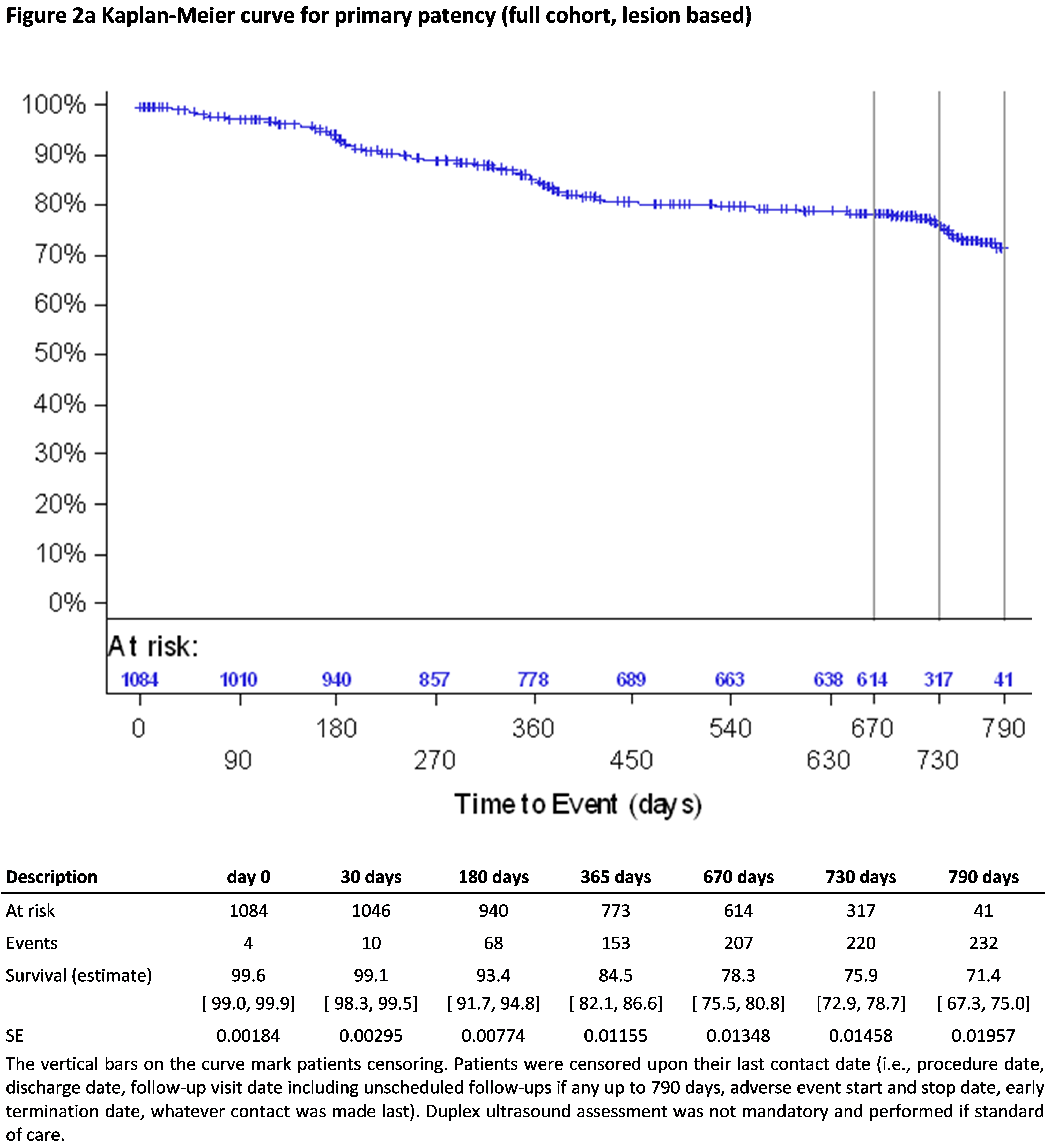

Supplement: Supplementary file 4 — Supplementary file4 (TIF 1407 kb) [file 270_2020_2663_MOESM4_ESM.tif]

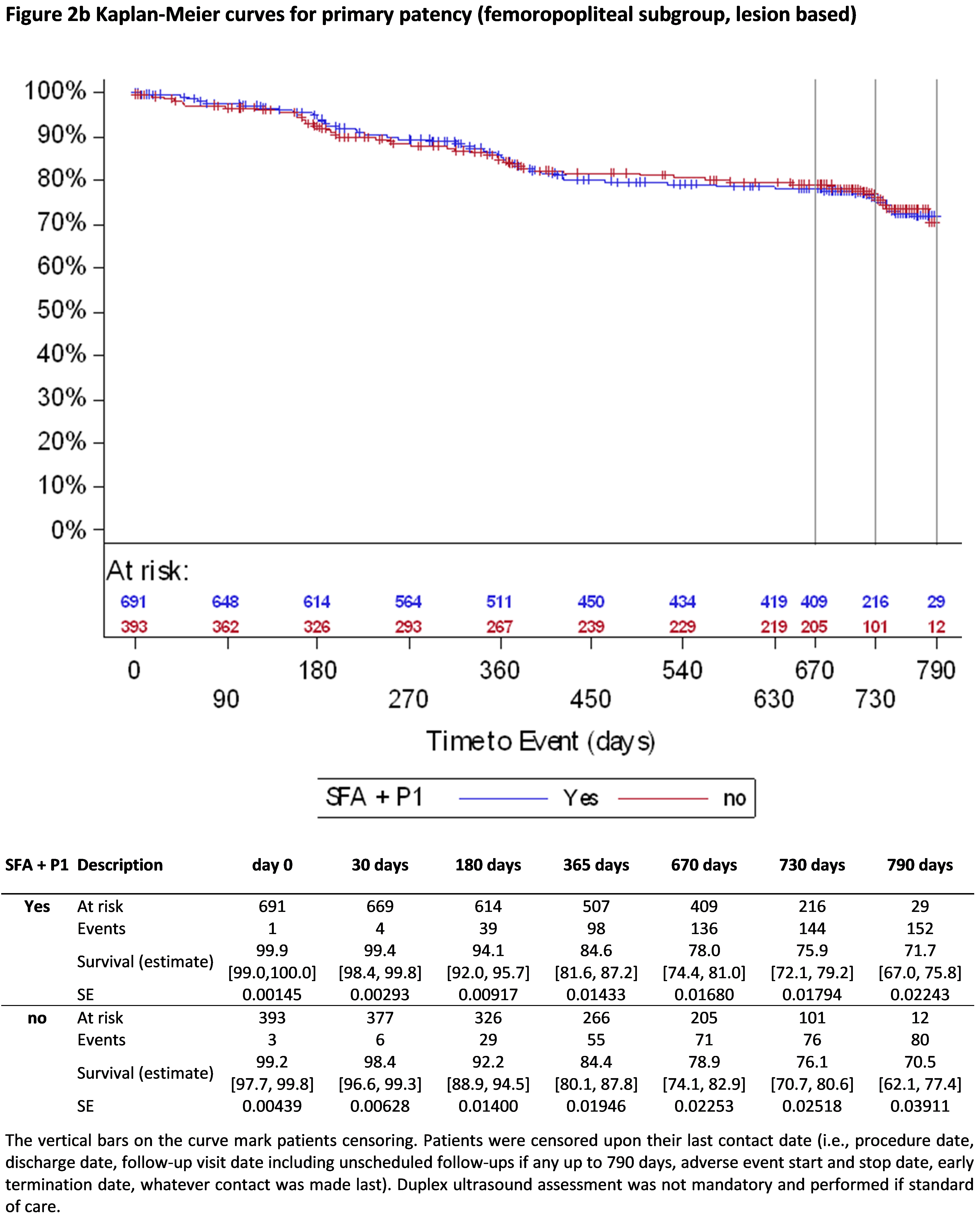

Supplement: Supplementary file 5 — Supplementary file5 (TIF 1772 kb) [file 270_2020_2663_MOESM5_ESM.tif]
